# Parity effect of bipolar quantum Hall edge transport around graphene antidots

**DOI:** 10.1038/srep11723

**Published:** 2015-06-30

**Authors:** Sadashige Matsuo, Shu Nakaharai, Katsuyoshi Komatsu, Kazuhito Tsukagoshi, Takahiro Moriyama, Teruo Ono, Kensuke Kobayashi

**Affiliations:** 1 Institute for Chemical Research, Kyoto University, Uji, Kyoto 611-0011, Japan; 2 Department of Physics, Osaka University, Toyonaka, Osaka 560-0043, Japan; 3 NIMS, WPI-MANA, Tsukuba, Ibaraki 305-0044, Japan

## Abstract

Parity effect, which means that even-odd property of an integer physical parameter results in an essential difference, ubiquitously appears and enables us to grasp its physical essence as the microscopic mechanism is less significant in coarse graining. Here we report a new parity effect of quantum Hall edge transport in graphene antidot devices with *pn* junctions (PNJs). We found and experimentally verified that the bipolar quantum Hall edge transport is drastically affected by the parity of the number of PNJs. This parity effect is universal in bipolar quantum Hall edge transport of not only graphene but also massless Dirac electron systems. These results offer a promising way to design electron interferometers in graphene.

Various parity effects, which mean that even-odd property of an integer physical parameter results in an essential difference, has been discovered in many physical systems, especially in mesoscopic physics. For example, the significance of the parity effect regarding the number of electrons is seen in the micron-sized superconducting island^1^, in the quantum dot in the Kondo regime^2^,^3^, and so on^4^,^5^,^6^. In case of quantum Hall (QH) edge transport, the electron spins are polarized in the edge state for odd filling factors, while they are unpolarized with even filling factors. This parity effect leads to polarizing nuclear spins^7^,^8^ and reading spin state in quantum dot^9^,^10^. In this way, the parity effects have played important roles on developing the research area.

Since graphene, which is monolayer film of graphite, was first isolated^11^,^12^, this wonder material has been studied extensively^13^. Especially, as electrons in graphene follow massless Dirac equation, the graphene is a nice platform to address universal features of the massless Dirac electron system, as is the case in the surface state of topological insulators^14^,^15^. In such systems, we can study the QH edge transport in bipolar regime, where the PNJs are formed as reported previously^16^,^17^. The QH edge states co-propagate along the PNJ and are uniformly mixed, reflected by a massless Dirac electron nature that a Landau level is formed at charge neutrality point. This bipolar QH edge transport is not realized on conventional two-dimensional electron gas in GaAs/AlGaAs heterostructure and there are only several experimental reports about the bipolar QH edge transport in graphene^17^,^18^,^19^,^20^,^21^,^22^,^23^ despite great interest.

Here we report a new parity effect of QH edge transport in graphene antidot devices with several *pn* junctions (PNJs). We study a graphene device with two types of regions in which the carrier types are *n* (electrons) and *p* (holes) which are arranged in series. The number of such regions is *M* + 1; therefore, the device has *M* PNJs. Furthermore, we place *N* antidots on all of the PNJs. The (*M*, *N*) = (2, 1) (even case) and (3, 1) (odd case) are schematically illustrated in Fig. 1(a,b), respectively. We calculated the conductance of graphene devices with such structures and discovered a significant parity effect of the resistance as a function of *N*, depending on parity of *M*. We implemented the proof-of-concept experiments and established the parity effect using a graphene device, whose optical picture is shown in Fig. 1(c). Our finding is a universal feature of bipolar QH edge transport in massless Dirac electron systems.

We first present parity effect found in the calculated results of the two-terminal resistance. We define *ν*_*tg*_ and *ν*_*bg*_ as the Landau level filling factors of the two types of regions tuned by both the top- and back-gate voltages and by only the back-gate voltage, respectively. When we calculate the resistance, we obtain the two-terminal conductance using the Landauer-Büttiker formula^24^ and then obtain the resistance as the inverse of the conductance. In this paper, we only consider the bipolar regime, sgn(*ν*_*tg*_) ≠ sgn(*ν*_*bg*_), which is unique to graphene (see the Supplemental Material for the unipolar regime). Our calculation treated here does not include possible interference effects.

We discuss the conductance in the even *M* case. We consider the filling factor in the regions connected to the source and drain electrodes, denoted by *ν*_*tg*_. We calculate the two-terminal conductance *G* as a function of *M, N* and when the filling factors *ν*_*tg*_ and *ν*_*bg*_ are ±2, ±6, 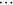 . The result is


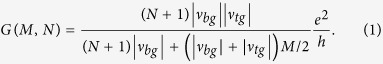


The resistance is obtained as *R* = *G*^−1^. As observed from this formula, the conductance increases as *N* increases. Interestingly, when *N* increases to infinity, the conductance becomes *ν*_*tg*_*e*^2^/*h*, namely, the conductance with no PNJs. Note that this representation is not symmetric with regard to the exchange of *ν*_*bg*_ and *ν*_*tg*_. Consequently, this means that the chirality of the QH edge states at the left and right sides are the same (see Fig. 1(a) for the case (*M*, *N*) = (2, 1).).

Next, we discuss the odd *M* case. In this case, we obtain the conductance as


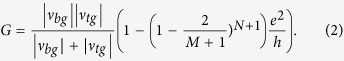


The resistance is obtained as *R* = *G*^−1^. The conductance increases as *N* increases, as found in the even *M* case. However, the conductance is quantitatively different from that in the even *M* case (Eq. (1)) in several aspects. First, the conductance dependence on *N* in the odd *M* case in Eq. (2) is expressed as a power of *N*, which is very different from the even *M* case in Eq. (1). In addition, we can obtain a peculiar result from Eq. (2), *ν*_*bg*_*ν*_*tg*_/(*ν*_*bg*_ + *ν*_*tg*_) · *e*^2^/*h*, as *N* approaches infinity. This conductance is the same as that observed in graphene with (*M*, *N*) = (1, 0). Furthermore, Eq. (2) is symmetric with regard to the exchange of *ν*_*bg*_ and *ν*_*tg*_. The physical laws possess charge, parity and time (CPT) reversal symmetry. From parity (mirror) symmetry, the conductance in odd *M* case is demanded to be symmetric about the exchange of *ν*_*bg*_ and *ν*_*tg*_, different from even *M* case. These unique properties in the bipolar regime originate from the difference in the chirality at the left and right (see the case (*M*, *N*) = (3, 1) in Fig. 1(b)). In particular, we note that Eq. (2) has a power-law dependence on the number of antidots *N*, which is only realised in bipolar QH edge transport.

These calculated results mean that the resistance formulas as a function of the number of antidots is strongly dependent on the parity of *M*, the number of PNJs. Such fundamental difference, the parity effect, should be useful for not only graphene but also QH devices of massless Dirac electron systems, such as the surface state of topological insulators^14^,^15^ and Dirac electrons in HgTe/CdTe heterostructure^25^. This parity effect is because of the chirality difference of the QH edge states around the antidots, which will be discussed later using an analogy to optical interferometers.

We implemented the following proof-of-concept experiments. While there are several reports regarding the transport in graphene PNJs in the QH regime^18^,^19^,^20^,^21^,^22^,^23^,^26^,^27^, there are no reports on devices with antidots and PNJs in the QH regime. We fabricated two types of graphene devices; one has an antidot, and the other has no antidot. In this article, we mainly discuss the results of the device with the antidot. We indicate the results of the device with no antidots in Supplemental Material. These devices were cleaved from a highly ordered pyrolytic graphite crystal via Scotch tape and transferred onto a Si substrate covered with 285-nm-thick SiO_2_. We estimated the thickness of graphene from an analysis of contrast in the optical pictures. Then, we etched the graphene samples using an oxygen plasma and created an antidot in graphene, as observed in the inset of Fig. 1(a). Subsequently, we pattered the electrodes using electron-beam lithography (EBL). Then, we deposited 5 nm of palladium and 30 nm of gold as the source and drain electrodes. Cross-linked PMMA was patterned by EBL on graphene as the insulating layer following the same method reported previously^28^,^29^. Finally, we placed two top-gate electrodes (3 nm of chromium and 30 nm of gold) on the insulating layer. We measured the two-terminal resistance of this graphene device at 2 K and 7 T. The graphene device, whose optical picture is shown in Fig. 1(c), has an antidot and two top-gate electrodes marked *α* and *β* in the inset of Fig. 1(c). We discuss the device for (*M*, *N*) = (1, 1),(2, 1), and (3, 1) by controlling the number of PNJs.

We begin with the (*M*, *N*) = (1, 1) case. We applied a top-gate voltage to only the electrode marked as *α* in Fig. 1(c) and measured the two-terminal resistance. Changing the top- and back-gate voltages corresponds to tuning *ν*_*bg*_ and *ν*_*tg*_. Figure 1(d) shows the device resistance as a function of the top-gate voltage *V*_*tg*_ and back-gate voltage *V*_*bg*_ at 2 K and 7 T. Cross sections of the image plot at *V*_*bg*_ = 15, 6, −4, and −13 V are shown in Fig. 1(e). The bipolar regime corresponds to the area in Fig. 1(d) surrounded by the green line marked as ‘*pn*’ and ‘*np*’. Several plateaus at which the resistance is almost constant exist in Fig. 1(d,e). We can decide the filling factors, *ν*_*tg*_ and *ν*_*bg*_, in bipolar regime from the resistance in unipolar regime. As shown in Fig. 1(d,e), for example, the device exhibits a resistance plateau of *h*/*e*^2^ in (*ν*_*tg*_, *ν*_*bg*_) = (±2, 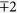), which is consistent with the calculated resistance from Eq. (2) with (*M*, *N*) = (1, 1) shown in Fig. 1(f). The resistances on the other plateaus of (*ν*_*tg*_, *ν*_*bg*_) are also consistent with our calculated results. This calculated result for (*M*, *N*) = (1, 1) is consistent with the resistance for the (*M*, *N*) = (1, 0) case as reported before^17^. The QH edge transport is not affected by the existence of the antidot in *M* = 1 case, so that we increase the PNJs to confirm the validity of Eq. (2).

We now discuss the device for the (*M*, *N*) = (2, 1) case (even case), where *pnp* and *npn* systems are realised for the condition of sgn(*ν*_*tg*_) ≠ sgn(*ν*_*bg*_). Experimentally, we applied the top-gate voltage *β* in Fig. 1(c). In Fig. 2(a), we plotted the resistance as a function of *V*_*tg*_ and *V*_*bg*_ in units of *h*/*e*^2^. The image plot is not symmetric with regard to the exchange of the vertical and horizontal axes. This property means that the conductance should be non-symmetric about the exchange of *ν*_*tg*_ and *ν*_*bg*_. Furthermore, we present the cross sections at *V*_*bg*_ = 15, 6, −4, and −13 V of Fig. 2(a) in Fig. 2(b). The resistance is different from the results for graphene *pnp* junctions without the antidot^18^,^19^,^26^ (see the Supplemental Material). For example, the largest resistance in our device is close to *h*/*e*^2^ in (*ν*_*tg*_, *ν*_*bg*_) = (±2, 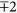) case, which is different from the predicted value of 3*h*/2*e*^2^ for graphene without the antidot. The calculated results for (*M*, *N*) = (2, 1) case in Fig. 2(c) not only in (*ν*_*tg*_, *ν*_*bg*_) = (±2, 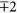) case but also the other plateaus are in good agreement with these experimental results in Fig. 2(a). This agreement means that Eq. (1) can well explain the QH edge transport with even *M* case on graphene antidots. Additionally, these experimental results in the unipolar regime are also consistent with the calculated results shown in the Supplemental Material.

Finally, we show the resistance for the (*M*, *N*) = (3, 1) case (odd case). We applied the same top-gate voltage to the two top-gate electrodes, *α* and *β*, in Fig. 1(a). Figure 3(a) shows the observed resistance dependence on *V*_*bg*_ and *V*_*tg*_. The image plot is symmetric with regard to the exchange of the vertical and horizontal axes; therefore, the conductance should be symmetric with regard to the exchange of *ν*_*tg*_ and *ν*_*bg*_. Figure 3(b) shows the cross sections at *V*_*tg*_ = 15, 6, −4, and −13 V of Fig. 3(a). These results also indicate a different resistance compared to the results of the device without an antidot (see Supplemental Material). For example, the resistance in (*ν*_*tg*_, *ν*_*bg*_) = (±2, 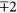) case is equal to 4*h*/3*e*^2^, which is different from the expected resistance, 2*h*/*e*^2^ without the antidot. Our calculated results in Fig. 3(c) demonstrate good consistency and provide an explanation of the experimental results. This experimental consistency validates the formula derived in Eq. (2) which explains the edge transport of graphene with an antidot and the three PNJs. Herein, our experimental study reveals the existence of the parity effect in the QH edge transport in graphene antidot devices in bipolar regime.

The confirmed parity effect in graphene antidot devices has a good analogy to optical systems, although the interference effect is not explicitly taken into account here. The conductance in the even case can be readily understood by comparing the Fabry-Perot interferometers (FPIs), which are constructed with two parallel beam splitters with reflectivity *r* > 0. The conductance dependence on *N* is consistent with the reflectivity, (*N* + 1)/((*N* + 1) + (1–*r*)/*r*), of FPIs arranged in series (not including the interference effect), which are constructed with *N* + 1 parallel beam splitters arranged in series (see the Supplemental Material). A schematic picture for the *N* = 1 case is shown in Fig. 4(b). Utilizing this analogy, we can regard PNJs between antidots as a beam splitter with *r* = |*ν*_*bg*_|/((*M*/2 + 1)|*ν*_*bg*_| + *M*/2|*ν*_*tg*_|) = *G*(*M*, 0)*h*/|*ν*_*tg*_|*e*^2^ and able to calculate the conductance representation without explicitly using the Landauer–Büttiker formula. In the graphene device, the edge states at the left (or right) side marked as ‘Input’, ‘Output1’, and ‘Output2’ in Fig. 4(a) correspond to the passing (or reflected) beam between the beam splitters marked as ‘Input’, ‘Output1’, and ‘Output2’ in Fig. 4(b).

In the odd case, we adopted Mach-Zender interferometers (MZIs) arranged in series, where two beams passing through a beam splitter of the MZI enter into the next beam splitter from different sides. This dependence with the condition of |*ν*_*tg*_| = |*ν*_*bg*_| can be viewed as the probability obtained at one port, (1–(1–2*t*)^*N* + 1^)/2, of *N* (see the Supplemental Material). *N* + 1 and *t* indicate the number of the beam splitters in the MZIs and the transmittance of the beam splitter, respectively. In condition with *ν*_*tg*_ = *ν*_*bg*_, *t* = 1/(*M* + 1) in (1–(1–2*t*)^*N* + 1^)/2 gives *G*(*M*, *N*)*h*/|*ν*_*tg*_|*e*^2^. A schematic picture of the *N* = 1 case is shown in Fig. 4(d). In this case, we can regard the edge states at the left and right, ‘Input’, ‘Output1’, and ‘Output2’ in Fig. 4(c) as the beams passing through the beam splitter, ‘Input’, ‘Output1’, and ‘Output2’ in Fig. 4(d).

As we discuss above, there are strong analogy between bipolar QH edge transport in graphene antidot device and optical interferometer. Thus, we believe that this system has a possibility to be a device for investigating interference effect of QH edge state in graphene. We ignored the interference effect between the edge states in our calculation and did not detect any interference pattern experimentally. This is because our device has not enough mobility so that the coherence length in the QH edge state is short. The higher quality graphene devices, such as graphene on h-BN substrate or suspended graphene will enable to measure the interference pattern.

In conclusion, we have studied the bipolar QH edge transport phenomena around graphene antidots with several number of PNJs. We derived the formulas to calculate the conductance of graphene with the antidots and PNJs in the QH regime. We experimentally confirmed these formulas with devices having one, two, and three PNJs. Consequently, we have established the parity effect that the conductance dependence on the number of antidots relies on the even--odd parity of the number of the PNJs. In particular, the odd *M* case is a unique transport regime realised on graphene antidot devices or other QH devices of massless Dirac electron systems. Furthermore, we suggest that this difference characterised by the parity can be understood by considering the analogy between the QH edge transport and the optical interferometers. Our results may be available to construct electron interferometer in graphene.

## Additional Information

**How to cite this article**: Matsuo, S. *et al.* Parity effect of bipolar quantum Hall edge transport around graphene antidots. *Sci. Rep.* **5**, 11723; doi: 10.1038/srep11723 (2015).

## Supplementary Material

Supplementary Information

## Figures and Tables

**Figure 1:**
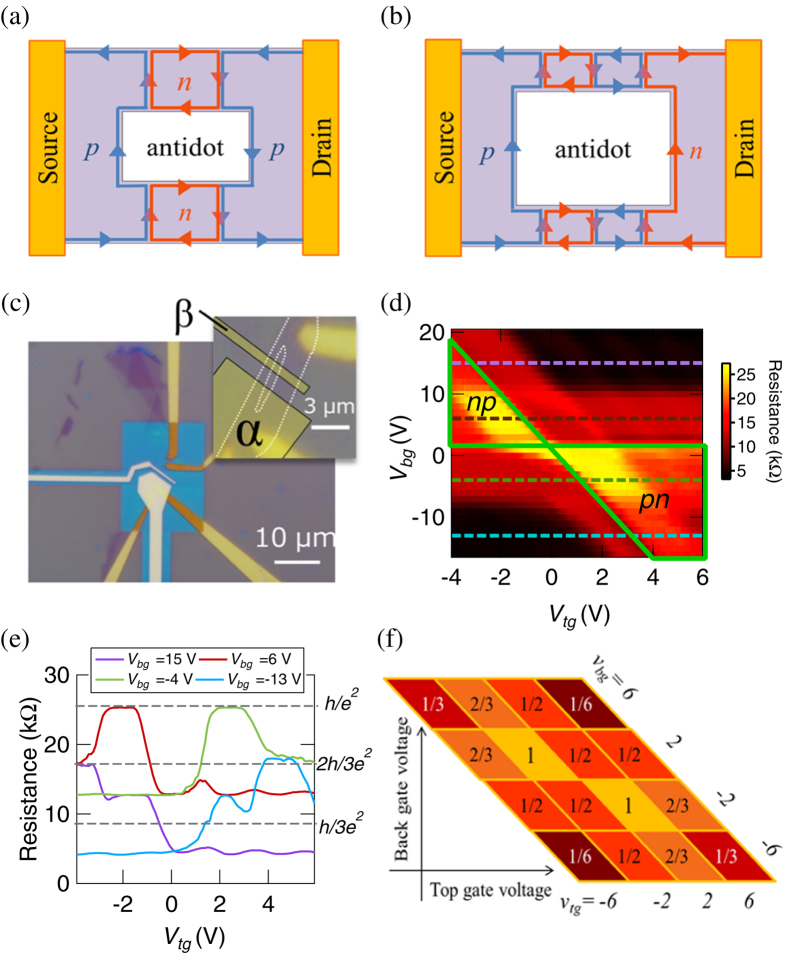
(**a**) Schematic picture of the chirality of the QH edge states around a single antidot with two PNJs, (*M*, *N*) = (2, 1) case. The chiralities of the edge states at left and right sides are the same in the even case. (**b**) Schematic picture of the chirality for the (*M*, *N*) = (3, 1) case. The chiralities of the edge states at left and right sides are different. (**c**) Optical image taken after fabrication of the top gate electrodes. Inset: Optical image taken before forming the insulating layer for the top gate structure. This device has a single open window (an antidot) as seen in the inset. We tuned the top gate voltages of these two top gate electrodes, marked as *α* and *β* in order to experimentally address the *pn*, *pnp* and *pnpn* junctions of graphene antidot. (**d**) Image plot of the resistance as a function of *V*_*tg*_ and *V*_*bg*_ for the (*M*, *N*) = (1, 1) case. The surrouded regions by the green lines are the bipolar regime (marked as “*pn*” and “*np*”). (**e**) The crosssection of the (**d**) at *V*_*bg*_ = 15, 6, −4, and −13 V. There are several plateaus, where the resistance is constant. (**f**) The calculated resistance for the (*M*, *N*) = (1, 1) case when the *ν*_*tg*_ and *ν*_*bg*_ are ±2, ±6, 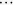 in unit of *h*/*e*^2^.

**Figure 2:**
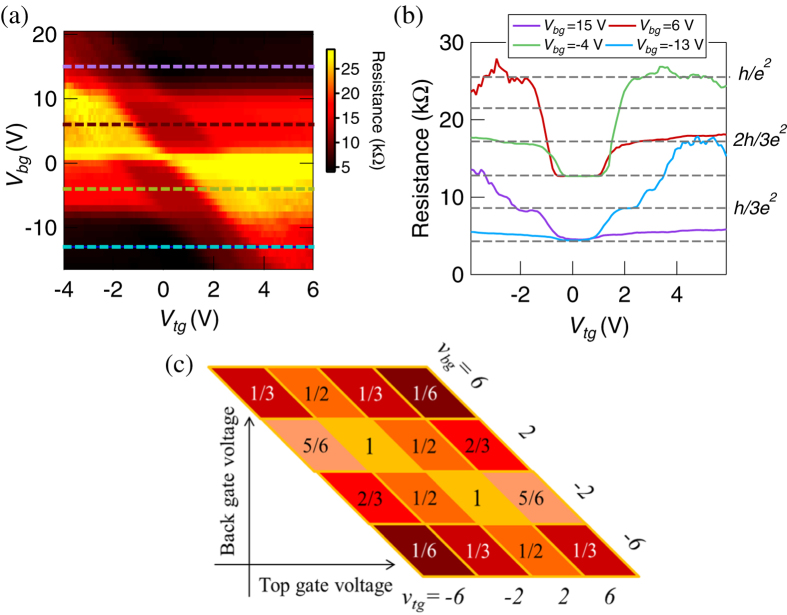
(**a**) Image plot of the resistance as a function of *V*_*tg*_ and *V*_*bg*_ for the (*M*, *N*) = (2, 1). This image is not symmetric with regard to the exchange of the vertical and longitudinal axises. (**b**) Crosssection of the (**a**) at *V*_*bg*_ = 15, 6, −4, and −13 V. (**c**) Calculated resistance when the *ν*_*tg*_ and *ν*_*bg*_ are ±2, ±6 in unit of *h*/*e*^2^. The experimental results of the plateaus are consistent with the calculated results shown in (**c**).

**Figure 3:**
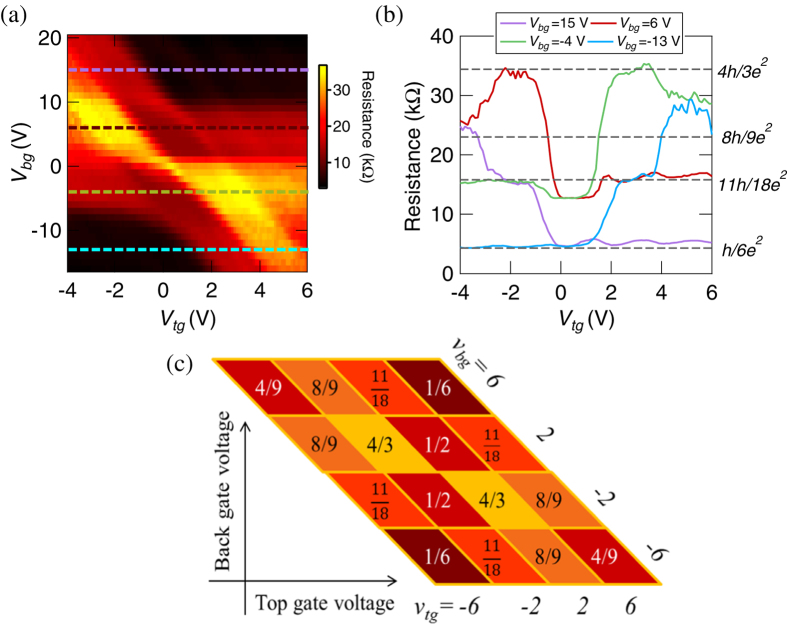
(**a**) Image plot of the resistance as a function of *V*_*tg*_ and *V*_*bg*_ for the (*M*, *N*) = (3, 1). This image is symmetric about the exchange of the vertical and longitudinal axises. (**b**) The crosssection of the (**a**) at *V*_*bg*_ = 15, 6, −4, and −3 V. (**c**) The calculated resistance when the *ν*_*tg*_ and *ν*_*bg*_ are ±2, ±6, 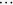 in unit of *h*/*e*^2^. The calculated results explain our experimental results well.

**Figure 4:**
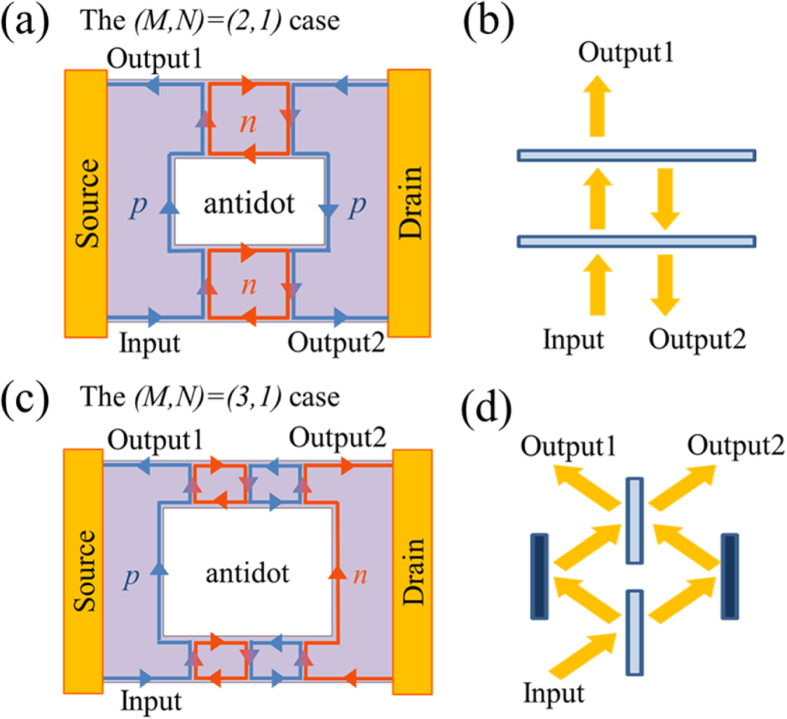
(**a**) Schematic picture of the QH edge transport in the (*M*, *N*) = (2, 1) case. Electrical current is injected from ‘Input’ and flow into ‘Output1’ and ‘Output2’. (**b**) Schematic picture of the FPI (for the *N* = 1 case). This system has the analogy to the even *M* case with *N* = 1 shown in (**a**). (**c**) Schematic picture of the QH edge transport in the (*M*, *N*) = (3, 1) case. (**d**) Schematic picture of the MZI (for the *N* = 1 case). This system has the analogy to the odd *M* case with *N* = 1 shown in (**c**). The different parity of *M* serves an different analogy of optical interferometers.
