# Supplementary material for: Parity effect of bipolar quantum Hall edge transport around graphene antidots
Source: Sci Rep. 2015 Jun 30;5:11723. doi: 10.1038/srep11723 (PMC4485210; doi:10.1038/srep11723)
Supplement: Supplementary Information [file srep11723-s1.pdf]

Supplemental Material: Parity effect of bipolar quantum Hall edge transport around graphene antidots

Sadashige Matsuo,[1,*] Shu Nakaharai,[2] Katsuyoshi Komatsu,[2] Kazuhito Tsukagoshi,[2] Takahiro Moriyama,[1] Teruo Ono,[1] and Kensuke Kobayashi[3]

[1]*Institute for Chemical Research, Kyoto University, Uji, Kyoto 611-0011, Japan*

[2]*NIMS, WPI-MANA, Tsukuba, Ibaraki 305-0044, Japan*

[3]*Department of Physics, Osaka University, Toyonaka, Osaka 560-0043, Japan*

Contents

Calculation of the conductance around graphene antidot 2

The conductance of the graphene device with an antidot in the unipolar regime 7

Optical interferometers 7

Experimentally observed resistance of graphene without the antidot 9

References 11

CALCULATION OF THE CONDUCTANCE AROUND GRAPHENE ANTIDOT

Here we show the derivation of the formulas of the conductance of graphene with antidots in quantum Hall (QH) regime. We assume that all the QH edge states along the *pn* junctions (PNJ) are mixed uniformly [1, 2]. We define filling factors in the region underneath top gate electrode and region that is not covered by the electrode as ν_{tg} and ν_{bg}, respectively. For simplicity, we restrict $\nu_{tg} > 0$ in this calculation. We define M and N as the number of the antidots and the junctions between two regions, respectively.

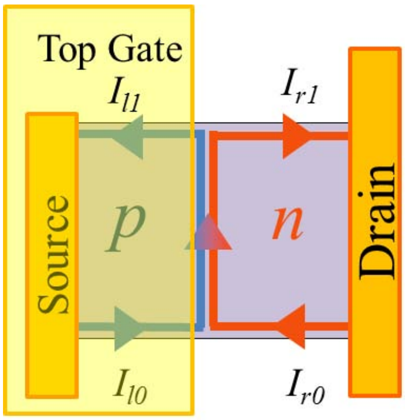

FIG. S1: Schematic diagram of the QH edge states in graphene with a single PNJ. We define I_{l0}, I_{r0}, I_{l1}, and I_{r1} as shown in this picture.

$(M, N) = (1, 0)$ **case** : For calculation of the conductance, we need to know the transmission probability of the current. We define I_{l0} and I_{l1} (I_{r0} and I_{r1}) as the current flowing in the region underneath top gate electrode (region that is not covered by the electrode) as shown in Fig. S1. The sign of the current is defined as the direction of the chirality in the region underneath the top gate electrode. Since we assume the uniform mixing, the relationship between these currents can be written as

$$I_{l1} = \frac{\nu_{tg}(I_{l0} - I_{r0})}{\nu_{tg} - \nu_{bg}}$$

$$I_{r1} = \frac{\nu_{bg}(I_{l0} - I_{r0})}{\nu_{tg} - \nu_{bg}}.$$

From these equations, we derive the following relations,

$$\Delta I_0 \equiv I_{l0} - I_{l1} = I_{r0} - I_{r1} = \frac{-\nu_{bg} I_{l0} + \nu_{tg} I_{r0}}{\nu_{tg} - \nu_{bg}}$$

$$I_{l1} = \frac{\nu_{tg}}{\nu_{bg}} I_{r1}.$$

They mean that the current difference between two edge states in one region, $(I_{l0} - I_{l1})$, is equal to the difference between the other region, $(I_{r0} - I_{r1})$. This property is available to calculate the conductance of the device with several antidots and the junctions.

$(M, N) = (2m, 0)$ **case** : We discuss the conductance of the device with $(M, N) = (2m, 0)$ case (m, positive integer). We enforce $|\nu_{tg}| < |\nu_{bg}|$ for simplicity. In this case, we define I_{l0} (I_{r0}) as the current injected from the source flowing in the leftmost region underneath top gate electrode (the current entering into the drain in the rightmost region underneath top gate electrode). The schematic picture of the QH edge states for the $(M, N) = (6, 0)$ case is shown in Fig. S2. We also define I_{l1} (I_{r1}) as shown in Fig. S2. Note that the filling factors of both sides connected to the source or drain electrode is the same in the $M = 2m$ case (namely, "p" in Fig. 2). For utilizing the property of $(M, N) = (1, 0)$ as discussed above, we define the current difference, ΔI_0, as $\Delta I_0 \equiv I_{l0} - I_{l1} = I_{r0} - I_{r1}$ and we have

$$I_{l0} = I_{r0} + m(1 - \frac{\nu_{tg}}{\nu_{bg}})\Delta I_0. \tag{1}$$

Since both sides are connected to the source or drain electrodes, $0 = I_{r0} - \Delta I_0$ holds. From these two relations, we obtain ΔI_0 described by I_{l0},

$$\Delta I_0 = \frac{\nu_{bg}}{(m+1)\nu_{bg} - m\nu_{tg}} I_{l0}$$

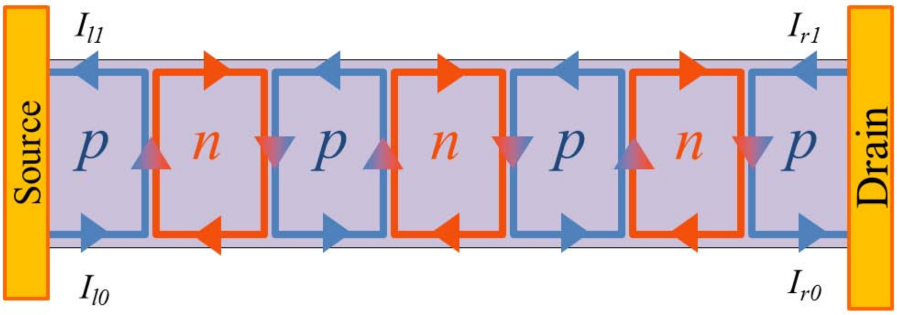

FIG. S2: Schematic diagram of the QH edge states in the device with six PNJs. We define I_{l0}, I_{r0}, I_{l1}, and I_{r1}.

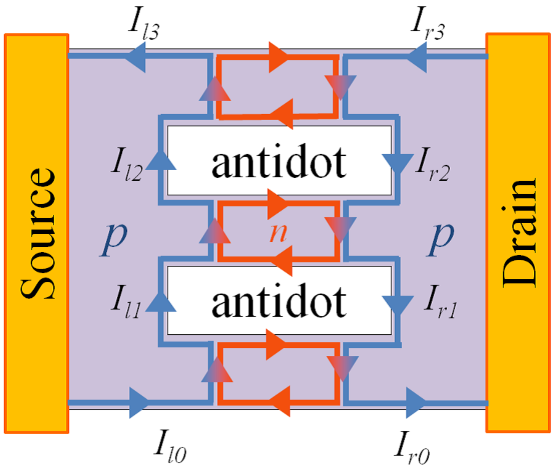

FIG. S3: Schematic diagram of the QH edge chirality in graphene for the $(M, N) = (2, 2)$ case. We define $I_{li}, I_{ri}(i = 0, 1, 2, 3)$ shown in this picture.

The transmission probability of the device is given by I_{r0}/I_{l0}; therefore the conductance, G, can be calculated from the Landauer-Büttiker formula as

$$G = \nu_{tg} \frac{e^2}{h} \frac{I_{ri}}{I_{li}} = \frac{\nu_{bg}\nu_{tg}}{(m+1)\nu_{bg} - m\nu_{tg}} \frac{e^2}{h}.$$

We note that this representation is consistent with the formula reported before [3].

$(M, N) = (2m, N)$ **case** We consider the $(M, N) = (2m, N)$ case. I_{li} and I_{ri} (i=1,2,...,N) are the current flowing along the antidot. The edge currents, $I_{l0}, I_{r0}, I_{l(N+1)}$ and $I_{r(N+1)}$, do not flow along the antidot but are running along the outmost boundary of the device. For example, we define I_{li} and I_{ri} $(i = 0, 1, 2, 3)$ for the $(M, N) = (2, 2)$ case as

shown in Fig. S3. The edge current, I_{l0} (I_{r0}), is injected from the source electrode (injected into the drain electrode). We define ΔI_i as $I_{l(i-1)} - I_{li} = I_{r(i-1)} - I_{ri}$. Now, according to Eq 1

$$I_{li} = I_{ri} + m(1 - \frac{\nu_{tg}}{\nu_{bg}})\Delta I_i. \tag{2}$$

In this case, we can describe $I_{li} = I_{l(i-1)} - \Delta I_{(i-1)}$ and $I_{ri} = I_{r(i-1)} - \Delta I_{(i-1)}$. Eq. 2 can be generalized into

$$I_{l(i-1)} = I_{r(i-1)} + m(1 - \frac{\nu_{tg}}{\nu_{bg}})\Delta I_i.$$

From this relation and Eq. 2 with converting i into $i-1$, we obtain

$$\Delta I_i = \Delta I_{(i-1)}.$$

We define this current difference as ΔI. Since $I_{r(N+1)} = 0$, $I_{l(N+1)} = m(1 - \frac{\nu_{tg}}{\nu_{bg}})\Delta I$ and $I_{l(N+1)} = I_{l0} - (N+1)\Delta I$ holds, we get $\Delta I = I_{l0}/(N+1-m(\nu_{tg}/\nu_{bg}))$. Since Eq. 2 with $i = 0$ gives the transmission probability, the conductance can be represented as

$$\frac{(N+1)\nu_{tg}\nu_{bg}}{(N+1)\nu_{bg} + m(\nu_{bg} - \nu_{tg})}\frac{e^2}{h}.$$

It is easy to show that this representation is valid for both bipolar and unipolar cases; namely the conductance in the bipolar regime is written as

$$G = \frac{(N+1)|\nu_{bg}||\nu_{tg}|}{(N+1)|\nu_{bg}| + m(|\nu_{bg}| + |\nu_{tg}|)}\frac{e^2}{h},$$

and the conductance in the unipolar regime is obtained as

$$\frac{(N+1)|\nu_{tg}||\nu_{bg}|}{(N+1)|\nu_{bg}| + m(|\nu_{tg}| - |\nu_{bg}|)}\frac{e^2}{h}.$$

$(M, N) = (2m-1, N)$ case Here, we discuss the $(M, N) = (2m-1, N)$ case. As well as the $M = 2m$ case discussed above, we define I_{li} (I_{ri}) as the current flowing along the antidot ($i = 1...N$) or the boundary ($i = 0$ or $N+1$).. For example, we show the $(M, N) = (3, 2)$ case as shown in Fig S4. The relationship between I_{li} and I_{ri} using Eq. 2 is written as

$$I_{li} = \frac{\nu_{tg}}{\nu_{bg}} I_{ri} + m(1 - \frac{\nu_{tg}}{\nu_{bg}})\Delta I_i. \tag{3}$$

Then, using $I_{li} = I_{l(i-1)} - \Delta I_{i-1}, I_{ri} = I_{r(i-1)} - \Delta I_{i-1}$ and Eq. 3 by replacing i with $(i-1)$, we obtained the recurrence relation of ΔI_i as

$$\Delta I_i = \frac{m-1}{m}\Delta I_{i-1},$$

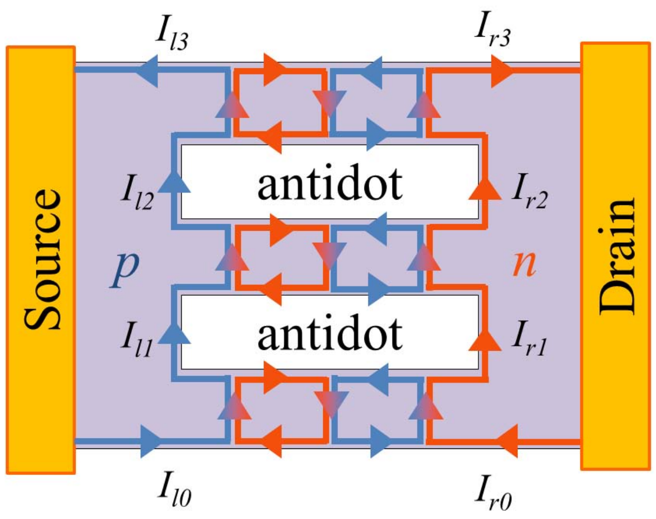

FIG. S4: Schematic diagram of the QH edge states in the device for the $(M, N) = (3, 2)$ case. We define $I_{li}, I_{ri} (i = 0, 1, 2, 3)$ shown in this picture.

therefore, we have $\Delta I_N = (\frac{m-1}{m})^N \Delta I_0$.

In the bipolar regime, namely, $\text{sgn}(\nu_{tg}) \neq \text{sgn}(\nu_{bg})$, the chirality of the edge states is opposite between I_{li} and I_{ri}. In this regime, $I_{r0} = 0$ holds. $I_{r(N+1)}$ satisfies

$$I_{r(N+1)} = \sum_{i=0}^{N} \Delta I_i.$$

Additionally, we get

$$\Delta I_0 = \frac{\nu_{bg}}{m(\nu_{bg} - \nu_{tg})} I_{l0}$$

From these relations, the transmission probability $I_{r(N+1)}/I_{l0}$ and hence, the conductance is obtained as

$$\frac{\nu_{bg}\nu_{tg}}{\nu_{bg} - \nu_{tg}} \Big(1 - (\frac{m-1}{m})^{N+1}\Big) \frac{e^2}{h}.$$

This is expressed as

$$\frac{|\nu_{bg}||\nu_{tg}|}{|\nu_{bg}| + |\nu_{tg}|} \Big(1 - (\frac{m-1}{m})^{N+1}\Big) \frac{e^2}{h}.$$

In the unipolar regime, $\text{sgn}(\nu_{tg}) = \text{sgn}(\nu_{bg})$, we should calculate I_{r0} as transmitted current into the drain electrode. $I_{r(N+1)}$ equals to 0, which is different from the bipolar regime. We calculate $I_{lN} = m(1 - \nu_{tg}/\nu_{bg})\Delta I_N - (\nu_{tg}/\nu_{bg})\Delta I_N$. From this relation, we have $I_{lN} = ((m-1)(1 - \nu_{tg}/\nu_{bg}) + 1)\Delta I_N$. We also have $I_{lN} = I_{l0} - \sum_{i=0}^{N-1} \Delta I_N$, therefore,

$$I_{l0} = m\Big(1 - \frac{\nu_{tg}}{\nu_{bg}}(\frac{m-1}{m})^{N+1}\Big)\Delta I_0$$

holds. From $I_{r0} = (\nu_{tg}/\nu_{bg})(I_{l0} - m(1 - \nu_{tg}/\nu_{bg})\Delta I_0)$, we can obtain the transmission probability, I_{r0}/I_{l0}. Consequently, the conductance is represented as

$$\frac{m^{N+1} - (m-1)^{N+1}}{|\nu_{tg}|m^{N+1} - |\nu_{bg}|(m-1)^{N+1}}|\nu_{bg}||\nu_{tg}|\frac{e^2}{h}.$$

The above calculation using the Landauer-Büttiker formula gives the conductance formulas in all the combinations of the filling factors. We complie these representations in Table I.

TABLE I: Conductance representations of the graphene device.

	Bipolar regime	Unipolar regime
$M = 2m$	$\frac{(N+1)\|\nu_{bg}\|\|\nu_{tg}\|}{(N+1)\|\nu_{bg}\|+m(\|\nu_{bg}\|+\|\nu_{tg}\|)}\frac{e^2}{h}$	$\frac{(N+1)\|\nu_{tg}\|\|\nu_{bg}\|}{(N+1)\|\nu_{bg}\|+m(\|\nu_{tg}\|-\|\nu_{bg}\|)}\frac{e^2}{h}$
$M = 2m-1$	$\frac{\|\nu_{bg}\|\|\nu_{tg}\|}{\|\nu_{bg}\|+\|\nu_{tg}\|}\left(1-\left(\frac{m-1}{m}\right)^{N+1}\right)\frac{e^2}{h}$	$\frac{m^{N+1}-(m-1)^{N+1}}{\|\nu_{tg}\|m^{N+1}-\|\nu_{bg}\|(m-1)^{N+1}}\|\nu_{bg}\|\|\nu_{tg}\|\frac{e^2}{h}$

THE CONDUCTANCE OF THE GRAPHENE DEVICE WITH AN ANTIDOT IN THE UNIPOLAR REGIME

We obtained the conductance representation in the unipolar regime as well. The calculated resistance from the representations complied in Table I are used in Fig.2(c), Fig.3(c), and Fig.4(c) of the main text. Our experimental results are consistent with the calculated resistance not only in the bipolar regime but also in the unipolar regime.

OPTICAL INTERFEROMETERS

Here we describe the transmission the properties of the Fabry-Perot Interferometers (FPI) and the Mach-Zehnder Interferometers (MZI).

First, we consider the series FPI, which consists of $N+1$ mirrors. The reflectivity and transmittance are represented as r and $t(\equiv 1-r)$. The $N = 2$ case is shown in Fig. S5(a). Then we define r_N and t_N as the reflectivity, "Output2" and transmittance "Output1" in

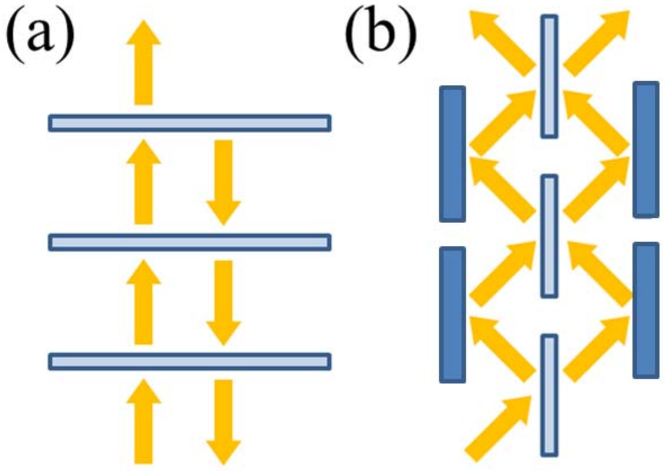

FIG. S5: (a)Schematic picture of the series FPIs with $N = 2$. (b)Schematic picture of the series MZIs with $N = 2$.

Fig.1(b) of the main text of the FPIs, respectively. The transmittance of the FPIs consisted of $N + 1$ mirrors is written as

$$t_{N+1} = t_N t + t_N r r_N t + t_N r r_N r r_N t + ... = \frac{t_N t}{1 - r r_N} \tag{4}$$

The inverse of the transmittance is given as

$$\frac{1}{t_N} = \frac{1}{t_{N-1}} + \frac{1}{t} - 1 = (N-2)(\frac{1}{t} - 1) + \frac{1}{t_2}$$

which is calculated from Eq. 4. From Eq. 4 and $t_2 = \frac{1-r}{1+r}$, the reflectivity is

$$r_N = \frac{N+1}{(N+1) + t/r}.$$

Next, we discuss the series MZI. This system consists of $N + 1$ of beam splitters, which split the beam into two paths. The $N = 2$ case is shown in Fig. S5(b). We consider r_N and t_N, which are the detected probability in the two ports, for example, the "Output1" and "Output2" shown in Fig.1(d) of the main text. The transmittance of the MZIs consisting of $N + 1$ mirrors is written as

$$t_{N+1} = t r_N + r t_N = 1 - r + (2r - 1) t_N. \tag{5}$$

Herein, solving this recurrence relation gives

$$t_N = \frac{1 - (1 - 2t)^{N+1}}{2}.$$

EXPERIMENTALLY OBSERVED RESISTANCE OF GRAPHENE WITHOUT THE ANTIDOT

We measured the graphene device without an antidot to confirm the quality of the graphene devices and uniform mixing of the QH edge state along the PNJ. The device without an antidot was fabricated by the same method as the device with an antidot.

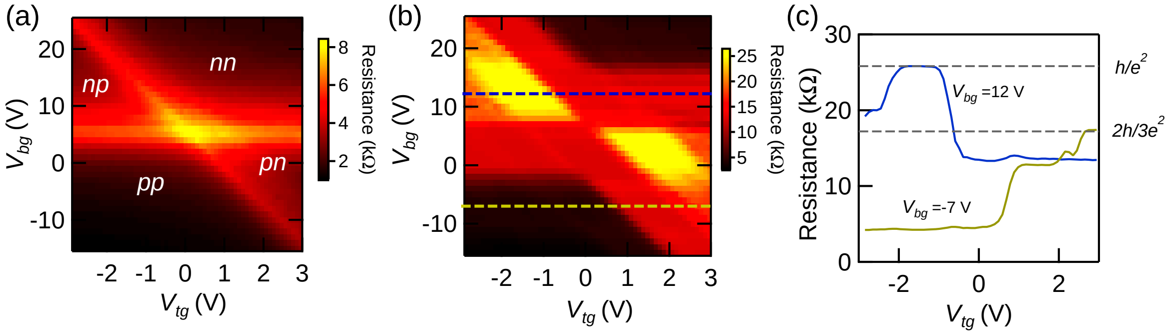

FIG. S6: (a)Image plot of the resistance of the device for the $(M, N) = (1, 0)$ case as a function of V_{tg} and V_{bg} measured at 2 K and 0 T. (b)Image plot of the resistance measured at 2 K and 7 T, for $(M, N) = (1, 0)$ case. (c)The crosssection of (b) at $V_{bg} = 12$ V (blue line) and $V_{bg} = -7$ V (yellow line).

We show the resistance as a function of top gate voltage (V_{tg}) and back gate voltage (V_{bg}) measured at 2 K and 0 T in Fig. S6(a). This corresponds to the $(M, N) = (1, 0)$ case. The image plot clearly indicates that we can control the carrier density of the two regions. Figure S6(b) and (c) are the image plot of the resistance measured at 2 K and 7 T and the crosssection of the image plot at $V_{bg} = 12$ V (blue line) and $V_{bg} = -7$ V (yellow line), respectively. These results are consistent with the theoretical result, $G = \nu_{bg}\nu_{tg}/(\nu_{bg} + \nu_{tg}) \cdot e^2/h$, which is obtained for the $(M, N) = (1, 0)$ case.

We also experimentally tested the $(M, N) = (2, 0)$ case. This case, the resistance as a function of V_{tg} and V_{bg} is shown in Fig. S7(a) and its crosssection is also shown in Fig. S7(b). The observed resistance is consistent with the calculated results shown in Fig. S7(c) as previously reported [3–5].

Additionally, we check the $(M, N) = (3, 0)$ case. Fig. S7(d) represents the resistance as a function of V_{tg} and V_{bg} and its crosssection is shown in Fig. S7(e). The resistance derived from our calculation are in Fig. S7(f) in unit of h/e^2. These results means that the uniformly

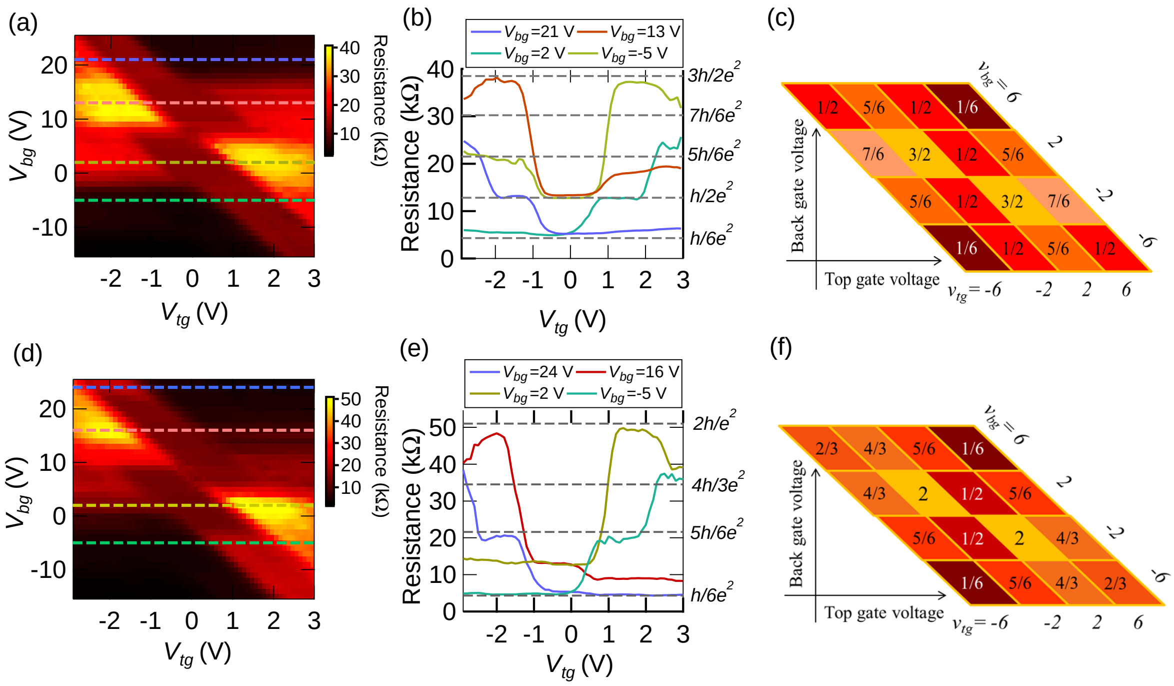

FIG. S7: (a)Image plot of the resistance of the device for the $(M, N) = (2, 0)$ case as a function of V_{tg} and V_{bg} tuned by the top gate electrode β. (b)The crosssection of the image plot (a) at $V_{bg} = 21, 13, 2,$ and -5 V. (c)The calculated resistance in unit of h/e^2 as a function of ν_{bg} and ν_{tg}. (d)Image plot of the resistance of the device for the $(M, N) = (3, 0)$ case as a function of V_{tg} and V_{bg} tuned by the top gate electrode α and β. (e)The crosssection of the image plot (a) at $V_{bg} = 24, 16, 2,$ and -5 V. (f)The calculated resistance in unit of h/e^2 as a function of ν_{bg} and ν_{tg}. This result explains our experimental results well.

mixing along the PNJ occurs in our device. This also supports that our graphene devices have enough quality to discuss the chirality control around the graphene antidots.

* Electronic address: matsuo@ap.t.u-tokyo.ac.jp

[1] Abanin, D. A. and Levitov, L. S. "Quantized transport in graphene p-n junctions in a magnetic field", Science **317**, 641 (2007).

[2] Williams, J. R., DiCarlo, L. , and Marcus, C. M. "Quantum hall effect in a gate-controlled p-n junction of graphene", Science **317**, 638 (2007).

[3] Özyilmaz, B. *et al.* "Electronic transport and quantum hall effect in bipolar graphene p-n-p junctions", Phys. Rev. Lett. **99**, 166804 (2007).

[4] Velasco Jr, J., Liu, G., Bao, W., and Lau, C. N. "Electrical transport in high-quality graphene pnp junctions", New Journal of Physics **11**, 095008 (2009).

[5] Velasco Jr, J. *et al.*, "Quantum transport in double-gated graphene devices", Solid State Communications **152**, 1301 (2012).
